# The association of meteorological parameters and AirQ+ health risk assessment of PM_2.5_ in Ratchaburi province, Thailand

**DOI:** 10.1038/s41598-022-17087-1

**Published:** 2022-07-28

**Authors:** Wissanupong Kliengchuay, Wechapraan Srimanus, Rachodbun Srimanus, Nuttapohn Kiangkoo, Kamontat Moonsri, Sarima Niampradit, San Suwanmanee, Kraichat Tantrakarnapa

**Affiliations:** 1 Department of Social and Environmental Medicine, Faculty of Tropical Medicine, Mahidol University, Bangkok, Thailand; 2 Environment, Health and Social Impact Unit, Faculty of Tropical Medicine, Mahidol University, Bangkok, Thailand; 3 School of Medicine, St. George’s University, True Blue, Grenada; 4 Graduate School of Environmental Development Administration, National Institute of Development Administration, Bangkok, Thailand; 5 W. Harry Feinstone Department of Molecular Microbiology and Immuniology, Johns Hopkins Bloomberg School of Public Health, Maryland, USA; 6 Department of Epidemiology, Faculty of Public Health, Mahidol University, Bangkok, Thailand

**Keywords:** Environmental sciences, Environmental social sciences

## Abstract

Air quality is heavily influenced by rising pollution distribution levels which are a consequence of many artificial activities from numerous sources. This study aims to determine the relationship between meteorological data and air pollutants. The health effects of long-term PM_2.5_ were estimated on expected life remaining (ELR) and years of life lost (YLL) indices in Ratchaburi province during the years 2015–2019 using AirQ+ software. Values obtained from the PM_2.5_ averaging, and YLL data were processed for the whole population in the age range of 0–29, 30–60 and over 60. These values were entered into AirQ+ software. The mean annual concentration of PM_2.5_ was highly variable, with the highest concentration being 136.42 μg/m^3^ and the lowest being 2.33 μg/m^3^. The results estimated that the highest and lowest YLL in the next 10 years for all age groups would be 24,970.60 and 11,484.50 in 2017 and 2019, respectively. The number of deaths due to COPD, IHD, and stroke related to long-term exposure to ambient PM_2.5_ were 125, 27 and 26, respectively. The results showed that older people (> 64) had a higher YLL index than the groups aged under 64 years. The highest and lowest values for all ages were 307.15 (2015) and 159 (2017). Thus, this study demonstrated that the PM_2.5_ effect to all age groups, especially the the elderly people, which the policy level should be awared and fomulated the stratergies to protecting the sensitive group.

## Introduction

The most detrimental factors that can influence the rise of air pollutants in urban cities are emissions from human activities^1^ such as power plants, industrial facilities, transportation, agriculture and waste treatment. In Thailand, there are three major sources of air pollution: vehicle emissions in the cities, biomass burning in rural and border areas,and emissions from industrial facilities^2^. Many research studies have found that there is a relationship between seasonal changes and their effects on air pollution. For example, the climate of summertime conditions contribute to the increase of particulate matter (PM_2.5_). Heat triggers more conditioning power usage in facilities and vehicles, thereby increasing particle emission, sunlight and heat transforms and worsen air pollution by triggering reactions between atmospheric particles such as nitrogen oxide and oxygen, forming ozone and transforming primary particles into even smaller particles which are hazardous to health. Heat waves and poor air movement create stagnant atmospheric conditions where air pollutants are trapped and accumulate over the ground level. Although indirectly, seasonal variations were significant contributors to human activities and air quality^3,4^. The studied by Ji et al. also demonstrated that heat waves enhance the impact of particulate matter on circulatory motality^5^. However, the temperature inversion also the remarkable factor that can trap the pollutant lead to higher pollutant concentration^6^. Additionally, winds influenced by seasonal variation can also affect air pollution by dispersing contaminants farther away from their source, nevertheless, higher winds in dry rural areas can generate dust^7,8^. The effects of these factors are shown clearly by a study in Beijing by Chen et al., and evidence has shown that seasonal changes are directly and indirectly associated with atmospheric pollution and air quality. During humid summer conditions, ozone concentration tends to increase with correlation to strong solar radiations, with intense photochemical reactions occurring atmospherically. Traffic during wintertime contributed to very high concentrations of nitrogen dioxide due to increased use of motor vehicles and coal combustion facilities, which produce atmospheric pollutants like sulfur dioxide and carbon monoxide. Wind fields have a crucial role in dispersing air contaminants particularly during heavily polluting conditions, with results suggesting that these dispersions mostly occurred during low wind speed circumstances (less than 1.5 m/s). Chen et al. have concluded that the winter and autumn were the seasons most polluted by PM_2.5_, PM_10_, SO_2_, NO_2_, and CO^9^. However, summer was most polluted by ozone as solar radiation-induced photochemical reactions and humid conditions contributed to the production of ozone during afternoons. Spatial patterns between air pollutants, where pollutant concentrations were much higher or lower in different regional areas of Beijing demonstrated the factor of areas with high levels of transportation. The correlation between wind profiles indicates that low wind speeds result in Beijing’s most heavily polluted incidences^9^. Thailand’s air pollution crisis results from 3 main sources: urban vehicle emissions, biomass burning, emissions from highly industrialized regions, and transboundary haze located in rural areas and borders^10^. Mueller applied risk estimates from the Global Exposure Mortality Model to calculate attributable mortality, and reported that 50,019 (95% confidence interval^1^: 42 189–57 849) deaths and 508 918 (95% CI 438 345–579 492) DALYs in 2016 was attributed to long-term PM_2.5_ exposure in Thailand^11^. Moreover, the studied in Thailand from Huang et al. mentioned the heatwaves related to mortality burden^12^.

Ratchaburi province is the one of Thailand’s central provinces, and is located west of Bangkok. This province is measured to be 5196 square kilometers wide with a population over Eight Hundred Thousand in 2018. The second contains the Tenasserim mountains and forests with an elevation of about 200–300 m. The last is the central area of this province is abundant with wetlands due to the river flowing through. Ratchaburi is one of the 14 provinces in Thailand with a large industrial estate. Industry here mainly deals with electricity, natural gas (Power plant), automotive, sugar, paper, and textiles, etc. The major commercial centers are distributed in the center of the province. Moreover, Ratchaburi is facing an air pollution crisis, due to also having a similar seasonal problem to Bangkok. The correlation between the varying air pollution data and varying seasonal changes in Ratchaburi, Thailand was drawn by analyzing statisticalresults collected from over a decade. In contrast to Beijing in China, Thailand undergoes three seasons that vary over each year; a dry season in mid-Febuary to mid- May, rainy/monsoon months from mid-May to mid-October, and a dry/cooler season during mid-October until mid-Febuary^13^. In the past years, several tools, including AirQ, BenMAP, Aphekom, and AirQ+ , and various organizations were developed for evaluating the health impacts along with the assessment of air pollution^14^. WHO developed the software AirQ+ for quantifying air pollution and its effect on health both in short-term and long-term. This research aims to survey the correlation between PM_2.5_ concentrations, meteorological parameters, and the estimate of all-cause annual mortality and mortality from cerebrovascular disease (stroke), ischemic heart disease (IHD), and chronic obstructive pulmonary disease (COPD) attributed to long-term exposure, and estimation of the health effects of PM2.5 on YLL indices in Ratchaburi from 2015 to 2019 using AirQ+ software.

## Results

### Air quality in Ratchaburi Province

The average concentration of PM_2.5_ was 26.86 μg/m^3^, which exceeding the NAAQS of Thailand (50 μg/m^3^) in some occasion, especially the dry season. However, severe amounts exceeding the WHO Air quality guideline values were also observed. The maximum 24-h average PM_2.5_ concentrations were 136.42 μg/m^3^, indicating that extreme particulate matter air pollution has occured in Ratchaburi province. CO, NO_2_ and SO_2_ concentrations in Ratchaburi were demonstrated to be under AQI standards. Ratchaburi had the O_3_ value at the minimum of 1.50 ppb, whereas the maximum concentration was 95.50 ppb. In some periods, the 8-h O_3_ presented over AQI. Additionally, the meteorological factor shows the lowest average wind speed (WS) being 1.2 m/s but the largest daily maximum would be 3.0 m/s. The average of Temperature (T) was 28.2 °C, and the highest of T was 33.2 °C. The annual average of wind direction (WD) blow from 245 degrees (Table 1).

**Table 1:** The annual parameters of air quality in Ratchaburi province with Thailand standard Air Quality Index.

Variable	Mean	SD	Variance	Minimum	Q1	Q3	Maximum	Standard
CO (8 h), ppm	0.60	0.19	0.04	0.24	0.45	0.70	1.14	9 ppm
NO_2_ (1 h), ppb	6.64	3.79	14.33	0.68	3.00	7.87	77.00	170 ppb
SO_2_ (1 h), ppb	1.42	1.74	3.04	0.09	0.61	1.87	47.27	300 ppb
PM_2.5_ (24 h), μg/m^3^	26.86	18.69	349.20	2.33	12.45	35.77	136.42	50 μg/m^3^
PM_10_ (24 h), μg/m^3^	46.84	25.40	644.94	7.08	28.40	58.52	167.21	120 μg/m^3^
O_3_ (8 h), ppb	35.33	21.77	474.08	1.50	19.03	49.95	95.50	70 ppb
Ws, m/s	1.20	0.35	0.12	0.28	0.94	1.42	3.02	
Wd, degree	245.0	39.0	1527.0	89.0	216.0	273.0	350.0	
Temp, °C	28.20	1.96	3.84	17.15	27.18	29.42	33.21	

Source: Pollution Control Department, Thailand.

### Correlation of PM_2.5_ and other air quality parameters

**Figure 1:**
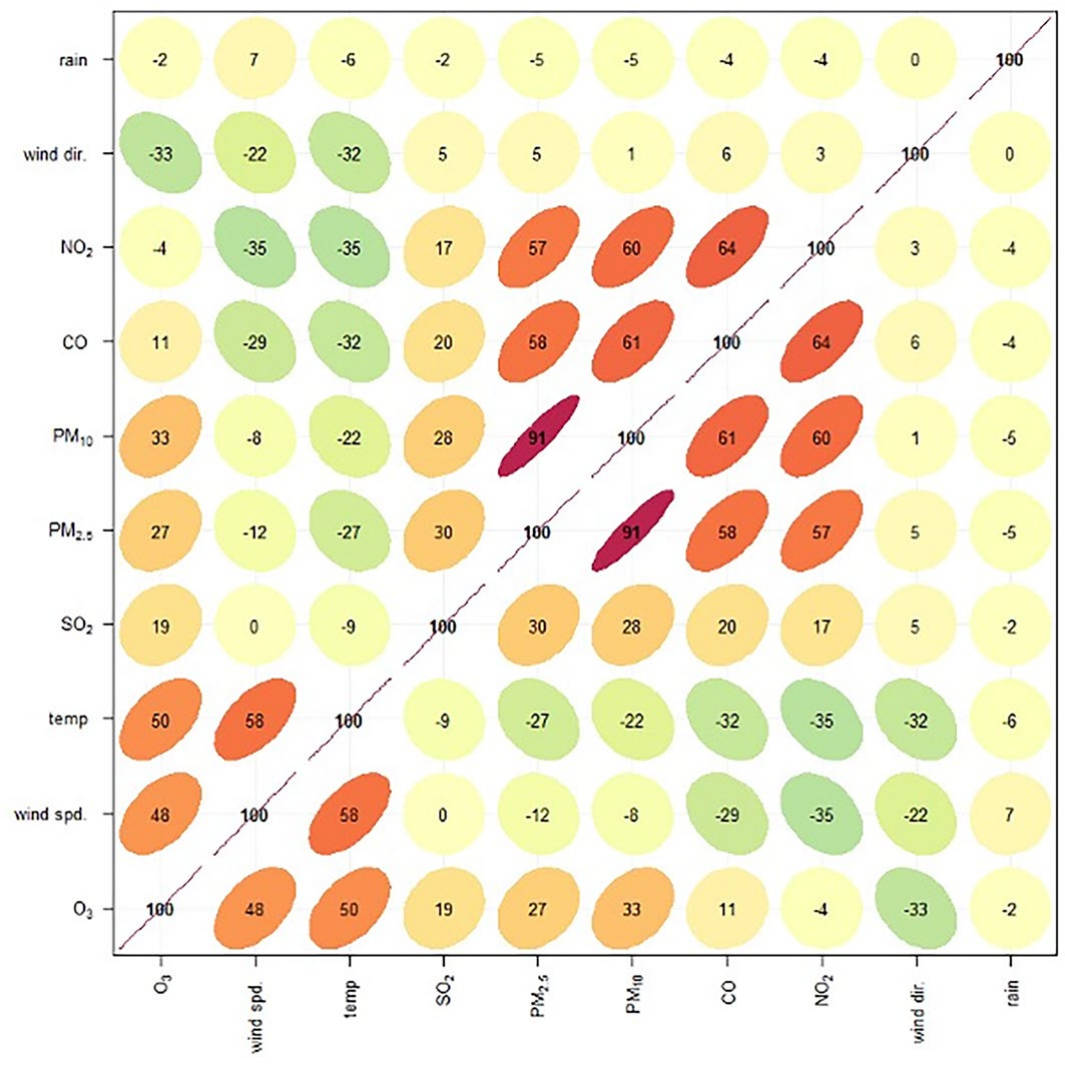
Spearman rank correlations between PM_2.5_ concentrations and levels of other air quality parameters.

The correlation between PM_2.5_ concentrations and the levels of PM_10,_ CO, NO_2_ SO_2_ and O_3_ demonstrated the correlation coefficients of 0.91, 0.58, 0.57, 0.30, and 0.27, respectively (p-value < 0.001). Hence, the PM_2.5_, PM_10_, CO, SO_2_, and NO_2_ concentrations are negatively correlated with Temperature and WS, shown by the correlation coefficient of − 0.27 and − 0.12, respectively (p-value < 0.001) (Fig. 1).

### Time series of PM_2.5_ in Ratchaburi province

**Figure 2:**
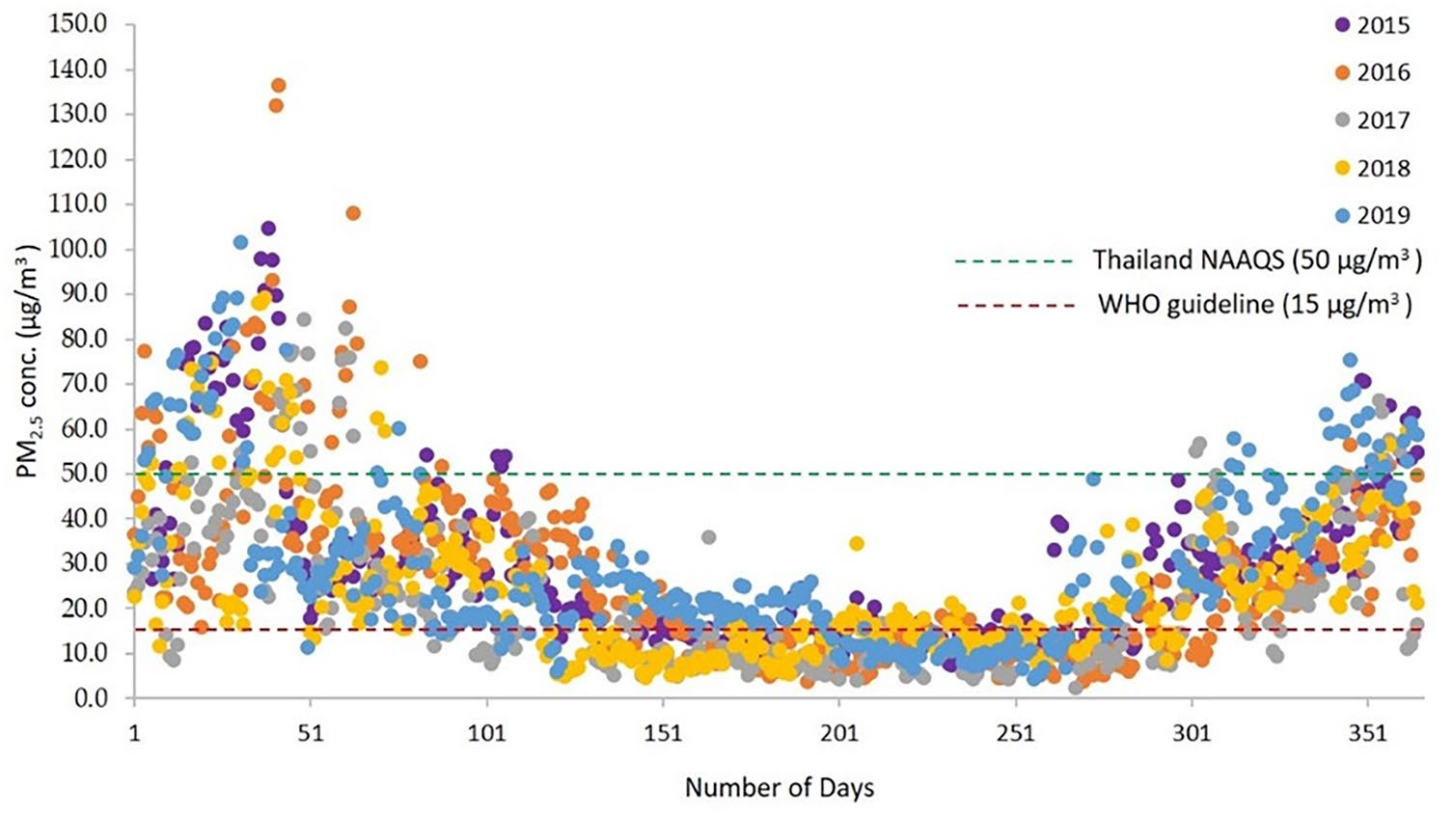
The pattern of PM_2.5_ concentration in Ratchaburi province during 2015–2019.

The PM_2.5_ concentration during 2015–2019 were presented in a similar pattern, the peak of 24 h concentration were observed twice a year, January to March and October to December as shown in Fig. 2. The annual average of PM_2.5_ concentration from 2015 to 2019 are shown in Table 1, along with the standard deviation (SD). In 2016, the highest PM_2.5_ concentration was 136.4 μg/m^3^, and the lowest was 2.3 μg/m^3^ in 2017. The annual concentration of PM_2.5_ and the amount of pollutants in the ambient air of Ratchaburi province was higher than the recommended value of WHO, and its variation ranged from 2.4 to 3.0 times higher than 10 μg/m^3^.

### AirQ+ mordel estimations

#### Chronic obstructive pulmonary disease (COPD), ischemic heart disease (IHD) and stroke attributable cases

**Figure 3:**
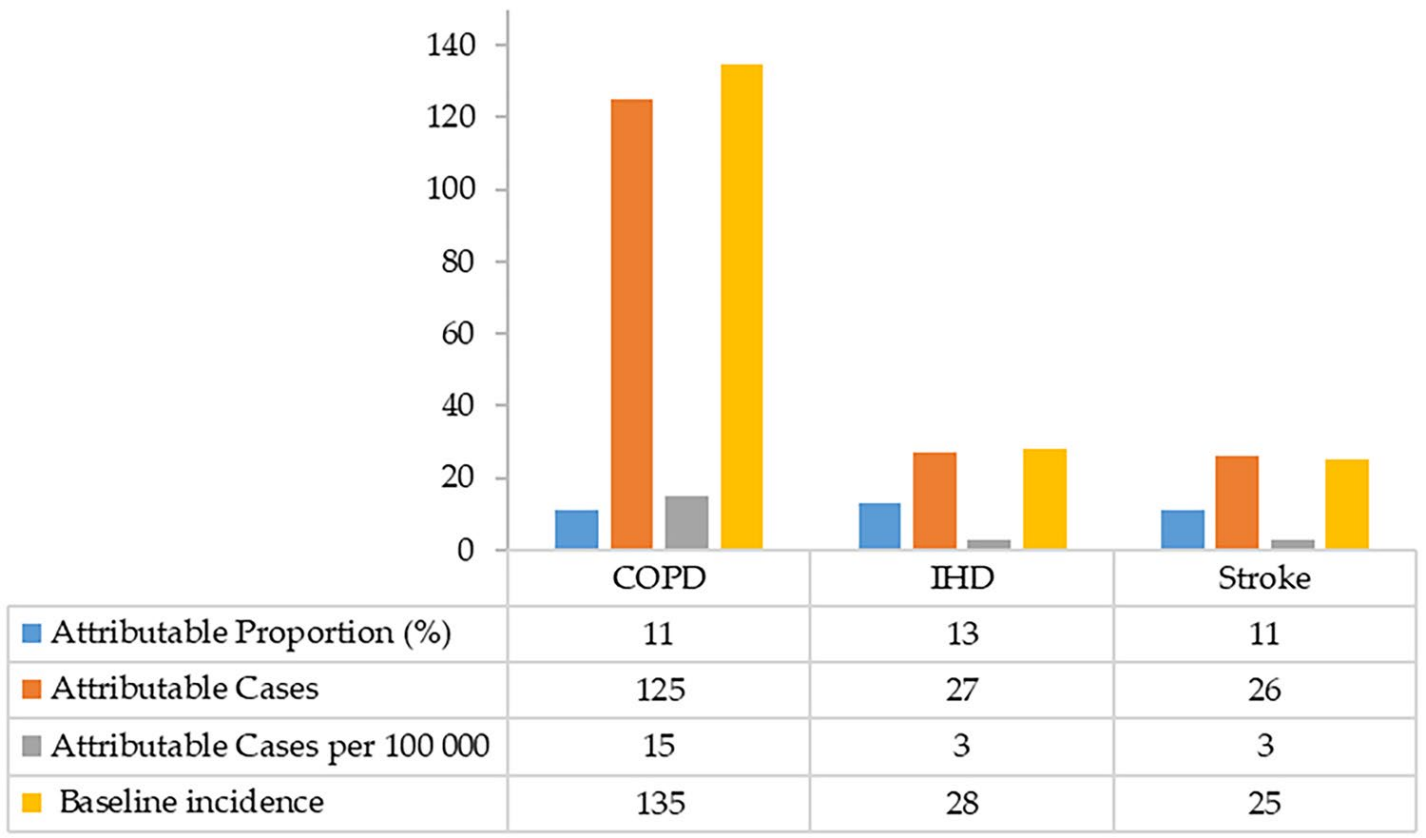
Estimation model of attributable proportion and attributable cases of endpoint mortality due to PM_2.5_ exposure in Ratchaburi province.

The AirQ+ estimation model demonstrated that people in Ratchaburi who have been exposed to long-term PM_2.5_ may have died due to COPD in the period of 2017–2018 for 125 cases as the annual average number. Moreover, the association beween PM_2.5_ and IHD was observed. The study found that 219 people may have died from IHD due to long-term exposure to PM_2.5_. In addition, there were 27 attributable cases per 100,000 for IHD due to PM_2.5_. Furthermore, the estimation model also demonstrated that 128 cases by an annual average may have died due to stroke-related complications due to long-term exposure to PM_2.5_. However, there were 26 attributable cases per 100,000 for stroke due to PM_2.5_ (Fig. 3).

#### Estimation of the health effects

The Year of Life Lost (YLL) was used as a health indicator in the development of public environmental policies^14^. Lin suggested that the essential to YLL can be used to identify appropriate interventions for risk management and improve the local efficacy for rapid response to air pollution^15^. Table 2 demonstrates the comparison between mortalies from PM_2.5_ between the years 2015 and 2019 in Ratchaburi Province. The highest YLLs were 243.6 years for the age of > 60 in 2019, and the lowest for the 0–29 age range is 5.1 years in 2017. The highest and lowest YLLs for all ages were 336.1 years in 2019 and 159.30 years in 2017, respectively. This health index was also examined for the next 10 years. The highest and lowest YLL in the next 10 years were 29,427.76 in 2019 for > 60 years age group and 476.60 in 2017 for agr range 0–29 years, respectively. Also, the highest and lowest values of this index were observed in the next 10 years for all ages: 31,819.90 in 2019 and 15,394.50 in 2017, respectively.

**Table 2:** The comparison of YLL because of deaths attributable to PM_2.5_ in Ratchaburi province.

Year	Age 0–29	Age30-60	Age > 60	All age	Over 10 years (age 0–29)	Over 10 years (age30-60)	Age > 60	Over 10 years (all age)
2015	8.5	76.2	221.2	305.9	851.4	7349.9	26,801.15	29,116.2
2016	8.1	72.7	210.9	291.6	811.8	7007.1	25,530.00	27,742.8
2017	5.1	43.2	111.1	159.3	476.6	4194.2	13,862.88	15,394.0
2018	6.5	67.6	188.2	262.3	606.5	6512.3	23,317.53	25,284.0
2019	9.6	82.9	243.6	336.1	883.6	7923.6	29,427.76	31,819.9

## Discussion

In this study, the air quality and health data from Ratchaburi province were collected as secondary data. The analysis to determining the association of PM_2.5_ to metheorological parameters was performed using AirQ+ software designed by World Health Organization^16^. The annual average of PM_2.5_ in Ratchaburi was 26.86 μg/m^3^, which could be observed as exceeding the standard in some period but extreamly exceeds the WHO guildline standard. The data was consistent with the study of Fold et al. on the exceeding PM_2.5_ concentration in central Thailand (Bangkok)^17^. In addition to the relationship with human activity, especially the automobile derived the PM pollution^18^. In terms of gaseous pollutants, the annual average concentration were observed under the AQI. Our study showed the weak positive correlation between PM_2.5_ and temperature and humidity, while a studied from China and Hong Kong showed the major meteorological factors affect the accumulation, dispersion and concentration of PM_2.5_^19,20^. From this point, it might be our sample size was small and the different in setting. The study of Vichit-Vadakan et al. showed that the gaseous pollutants i.e. nitrogen, nitric oxide and ozone were strong associated with several different mortality outcome^21^. Over the course of five years’ data collected, we detected the same pattern of PM_2.5_ concentration in every collected years with two peaks occurring twice a year in dry season that comply with previous studied that observed the PM_2.5_ obsvious exceed the standard in dry season^22^. The studied of Figueroa-Lara et al. showed that the PM_2.5_ concentration in warm-dry season were significantly higher than cold-dry season^23^. The AirQ+ estimation of health effect demonstrated The highest and lowest YLLs for all ages were 336.1 years in 2019 and 159.30 years in 2017 and for the next 10 years. Furthermore, the highest and lowest values of this index were observed in the next 10 years for all ages: 31,819.90 in 2019 and 15,394 in 2017. The result of this study was consistent with Zallaghi et al. on the health effects of long-term exposure to PM_2.5_ on ELR and YLL in Ahvaz, Iran and Zhu et al. on the effect of ambient air pollution load on YLL in Wuxi, China, using a nonlinear model (2012–2015)^14,24^. Moreover, the study site from China, Japan and South Korea also found the trend of total deaths were driven by population aging^25^. On the other hand, the study was inconsistent with Guo et al. study on the air pollution load on the YLL in Beijing, China^26^. However, when compared with studied from Middle Eastern populations, our study showed the YLL was lower dramatically^27^. Nevertheless, the different setting like in Europe, the overall air pollution that relate to morbidity and mortality have decrease considerably in the last three decades^28^. This study also showed the elderly population have high YLL value than others age group, due to the sensitive group. Consequently, the aging population might impact from medical resources and expenditures, and diseases by age group^29^.

This study was performed as a pilot study to investigating the PM_2.5_ effect on YLL in the western side border of Thailand. The results of this study could potentially be beneficial as platforms for further studies in other areas, provided with valid data, hence, convincing the environmental health department on the concern of the effects of particulate matter and pollutant parameters that could pose signifcant impacts on health.

## Conclusions

This study aimed to evaluate the relationships of PM_2.5_ with meteorological in Ratchaburi province. The results of correlation analysis showed a weak positive correlation between PM_2.5_ concentrations and average monthly temperature (r = 0.42, P < 0.05) and average monthly humidity (r = 0.37, P < 0.05) in Ratchaburi province. For this purpose, AirQ+ as the health effects of long-term exposure to PM_2.5_ on YLL for 10 years. In dry period, PM_2.5_ concentration was over the WHO standard amount in all studies years. The results also showed that older people (> 60) had a higher YLL index than those group aged under 60 years. The highest and lowest values for all age were 336.10 (2019) and 159.30 (2017). On the other hand, the highest and lowest rates of this index were observed in the next 10 years for all ages: 31,819.90 in 2019 and 15,394 in 2017, respectively. The finding of this research can be used for further air quality and exposure measurement for reducing mortality.

However, the core limitation of this study is small sample size; the observed trend might not represent the overall population of the west part of country. The larger sample size and multi-area study should be considered to observing and verifying the trends. This study could be platform for research to increase the study scale, leading to environmental policy, health plan evaluation, as well as the welfare plan for population.

## Methods and data collection

### Study site

**Figure 4:**
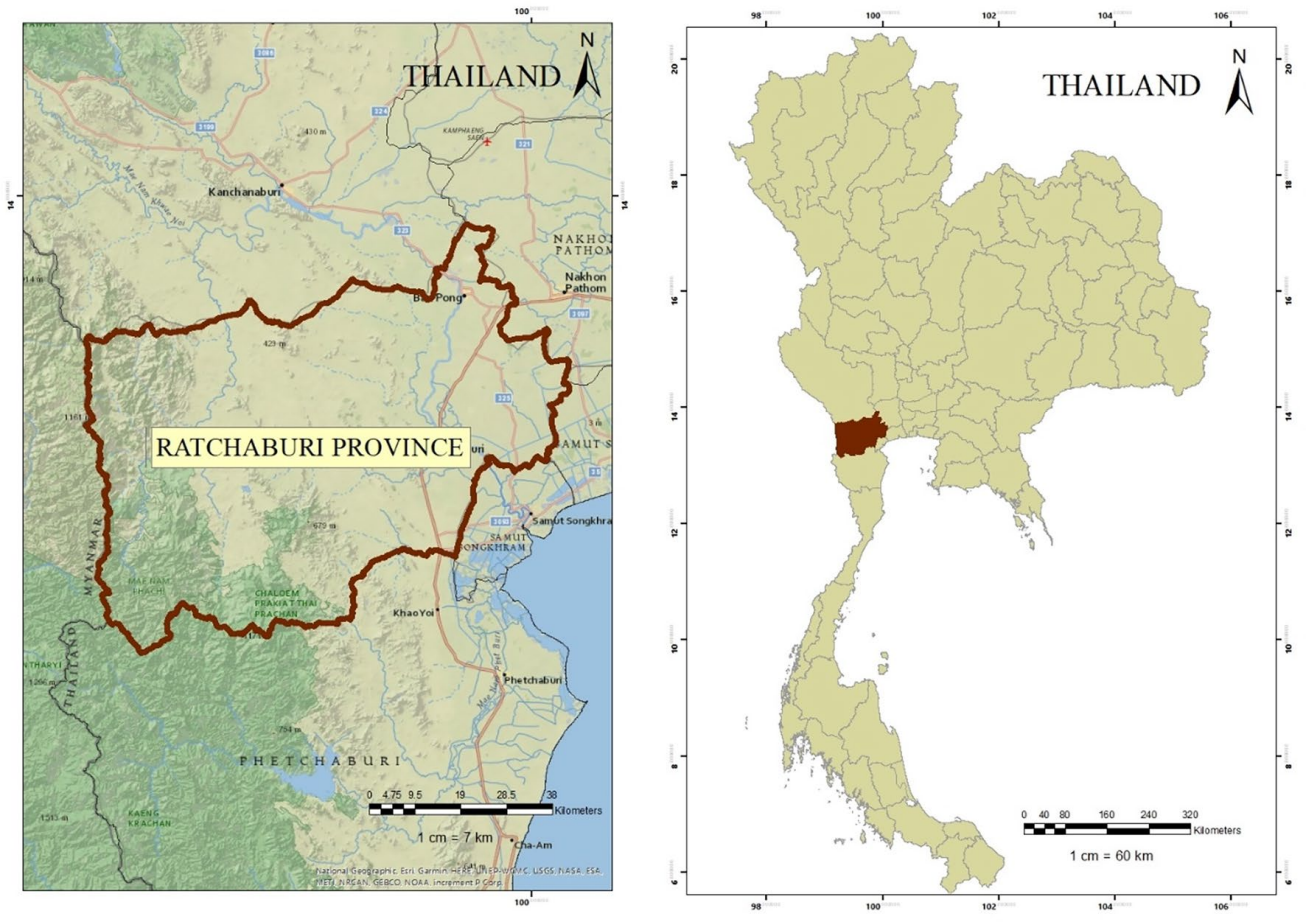
Location of sampling site in Ratchaburi province, Thailand.

Air monitoring stations were positioned in Ratchaburi, located at the Regional Environment Office 8th. Ratchaburi province lies in Western Thailand and its border is connected to the Tanintharyi Region of Myanmar. The total population is over Eight Hundred Thousand and the area is 5196 square kilometers (Fig. 4). This city is suffering from air pollution from several sources such as powerplants, industry, agricultural activities, and transportation.

### Data collection

The PM_2.5_ and air quality parameters data were collected in the period of January 1st, 2015 to December 31st, 2019 from Pollution Control Department (PCD). The data was acquired at every hour from 1 am. to 11 pm. The data cleaning process was performed by excluding the invalid time and date. The health data in this study was collected in the period of 2015 to 2019 with the ICD10 code of J44 for Chronic Obstructive Pulmonary Diseses (COPD), I2 for Ischaemic Heart Disease (IHD) and I6 for stroke recorded by Ministry of Public Health, Thailand.

### AirQ+ software

The valid PM_2.5_ and health data for over a 5 year period were entered into the AirQ+ software to calculate the YLL. The baseline incidence rate of all-cause mortalities and the averages data of PM_2.5_, Health data and YLL were converted to a .CSV file and inputs to AirQ+ software^14^. Table 3 shows a summary of inputted data to the AirQ+ software.

**Table 3:** Data input to AirQ+ software.

Year	PM_2.5_ (μg/m^3^)	Population of Ratchaburi Province	Population of all-cause mortality
2015	28.3 ± 19.0	860,550	6017
2016	27.1 ± 20.2	868,853	6286
2017	23.9 ± 18.0	870,769	5979
2018	25.0 ± 16.8	872,616	6185
2019	29.5 ± 18.8	873,310	6596

### Statistical analysis

The descriptive statistic (mean, standard variation and varience) were used to analyze the annual air quality parameters to compare with the standard of Air Quality Index (AQI). The spearman rank correlation was performed to investigate the correlation of PM_2.5_ to other air quality parameters.

### Ethics approval and consent to participate

The study was approved by the Ethics Committee of Faculty of Tropical Medicine, Mahidol University TMEC 21-055 in compliance with Declaration of Helsinki, ICH guildline for Good Clinical Practice and other international Guildlines for Human Research Protection. Inform Consent is not applicable for this study due to the secondary data was obtained.

## Data Availability

Data used and analyzed in the manuscript will be made available upon request to corresponding author.
